# Ultrafast microwave synthesis of defect-rich graphitic carbon nitride for enhanced photocatalytic degradation of procaine

**DOI:** 10.1039/d6ra03829h

**Published:** 2026-07-03

**Authors:** Debora Briševac, Floren Radovanović-Perić, Kristina Tolić Čop, Gordana Matijašić, Lidija Ćurković, Davor Ljubas

**Affiliations:** a Faculty of Mechanical Engineering and Naval Architecture, University of Zagreb Ivana Lučića 5 Zagreb Croatia debora.brisevac@fsb.unizg.hr lidija.curkovic@fsb.unizg.hr davor.ljubas@fsb.unizg.hr; b Faculty of Chemical Engineering and Technology, University of Zagreb Marulićev trg 19 Zagreb Croatia fradovano@fkit.unizg.hr ktolic@fkit.unizg.hr gmatijas@fkit.unizg.hr

## Abstract

Novel defect-abundant graphitic carbon nitride photocatalysts (g-C_3_N_4_–CN) were successfully prepared *via* rapid and energy-efficient direct microwave synthesis performed exclusively in a household microwave oven, representing an unconventional and rarely explored approach compared to conventional thermal synthesis in an electrical furnace. Six samples were synthesized from urea at magnetron powers of 600 and 800 W for 10, 12, and 15 min, resulting in drastically shorter synthesis times and reduced energy consumption compared to thermal synthesis. A comprehensive characterization was conducted, including FTIR, XRD, XPS, DRS, SEM, and BET analysis. In addition, photocatalytic tests were conducted under UV-A, visible, and simulated solar light to determine CN's ability to degrade procaine, a pharmaceutical primarily used as a dental anesthetic. Degradation efficiency, kinetic parameters, and photocatalytic mechanisms were determined and analyzed. The analysis revealed the formation of a highly amorphous carbon-rich CN structure with abundant structural imperfections, thereby enhancing visible-light photocatalysis. The main objective of this research was not only to achieve a substantially shorter synthesis time compared to conventionally prepared CN in an electrical furnace, but also to obtain a photocatalyst with superior activity under visible light. Among the prepared samples, the CN sample, synthesized at 800 W for 15 min, achieved the highest photocatalytic performance with 72.4% procaine degradation within 120 min under visible light, compared to only 35.4% for the conventionally prepared CN sample. Furthermore, the scavenger studies demonstrated that superoxide radicals (˙O_2_^−^) are the most influential in the degradation process. Overall, this work provides insight into many possibilities for CN synthesis, offering new opportunities for efficient visible-light-driven removal of pharmaceutical pollutants.

## Abstract 2

Defect-rich amorphous graphitic carbon nitride prepared within minutes in a household microwave oven exhibits strongly enhanced visible-light-driven degradation of procaine, highlighting a sustainable photocatalyst synthesis.

## Graphical abstract

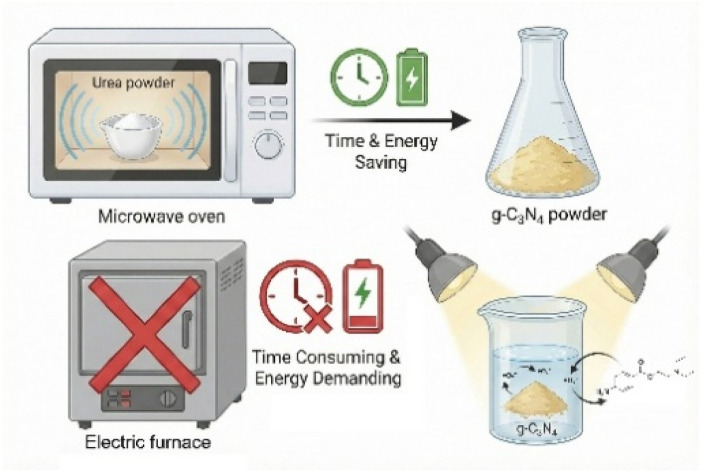

## Introduction

The contamination of freshwater resources by pollutants has become a critical environmental challenge worldwide. Advanced nanotechnologies and structural modifications of nanomaterials have gathered significant attention in the environmental remediation field, particularly for the elimination of persistent organic micropollutants (OMPs) from wastewater.^1^ Conventional wastewater treatment technologies are often insufficient for the complete removal of OMPs, including pharmaceuticals, pesticides, industrial chemicals, and personal care products, which can be detected in aquatic environments in trace amounts (ng L^−1^ to µg L^−1^).^2^ Despite the low concentration of OMPs, these compounds pose a significant risk to the ecosystem and human health due to their persistence, bioaccumulation, and continuous release in water bodies.^3^ Therefore, it is necessary to develop an efficient and sustainable strategy for the elimination of emerging contaminants.

Among the available approaches, advanced oxidation processes (AOPs), in particular, heterogeneous photocatalysis, have attracted significant attention as a promising method for OMP degradation.^4^ The photocatalytic process consists of light activation of a semiconductor material, resulting in generation of reactive oxygen species (ROS), such as superoxide anions and hydroxyl radicals, which participate in decomposition of pollutants into less harmful products.^5^ However, many of the commonly used semiconductors can only be activated by UV irradiation. From a practical perspective, optimizing photocatalysts for visible light utilization is crucial because visible light accounts for almost 50% of the solar spectrum reaching the Earth's surface, whereas UV light constitutes less than 5%. For real-world and water-treatment applications, usage of visible spectrum is far more important than relying on strictly UV fraction.^6–8^

Graphitic carbon nitride, CN, has emerged as a highly attractive metal-free material due to its chemical stability, low cost, environmental friendliness, and suitable physical properties.^9,10^ With a band gap of ∼2.7 eV, it can be activated under visible light irradiation, enabling ROS generation for a wide range of applications, including water splitting, CO_2_ reduction, and pollutant degradation.^11–13^

Typically, CN is synthesized *via* thermal polymerization of carbon- and nitrogen-rich precursors such as urea, melamine, thiourea, or dicyandiamide in conventional electric furnaces, which require long reaction times and high energy input.^14,15^ Therefore, alternative synthesis methods that reduce processing time are of high interest. A novel approach has been found in microwave synthesis due to its rapid heating and energy efficiency.^16^

**Fig. 1:**
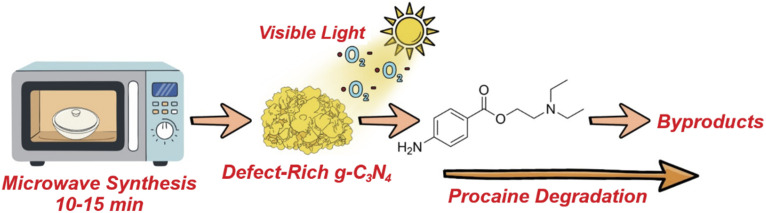
Schematic illustration of unconventional direct synthesis of defect-rich g-C_3_N_4_ (CN) in a household microwave oven.

Development of energy-efficient synthesis methods, such as microwave-assisted approach, makes a crucial step toward addressing the techno-economic requirements for scaling up photocatalytic processes to industrial level.^17^ In contrast to conventional electrical furnace synthesis, the microwave-assisted approach works on a completely distinct physical heating mechanism – volumetric dielectric heating. When exposed to microwave irradiation, polar molecules within the precursor matrix undergo rapid dipole rotation and intramolecular friction. That causes an ultra-fast heating rate and generates uniform thermal distribution from the core of the sample outward (inside-out heating).^18^ The challenge occurs in the low microwave absorbing ability of graphitic carbon nitride precursors, limiting the controlled synthesis. To overcome this, microwave susceptors such as graphite, silicon carbide, and metal oxides have been introduced to precursors to enable efficient heat transfer and rapid polymerization.^19^ However, such precursors could potentially be difficult to remove from the final product and can interfere with the polymerization process. While recent reports have demonstrated the ability to synthesize CN photocatalysts using microwave-assisted methods, studies employing a simple household microwave reactor remain scarce in the literature (Table 1). While CN has been widely investigated for the removal of pharmaceutical contaminants, to the authors' knowledge, no studies have reported the degradation of procaine. Procaine has been detected in pharmaceutical wastewater and wastewater treatment plants' effluents at concentrations around 1 µg L^−1^.^20^ Additionally, surface defect engineering has been recognized as one of the most effective strategies in improving overall photocatalytic performance of g-C_3_N_4_ materials.^17^ Therefore, this work focuses on describing a rapid and energy-efficient microwave synthesis of defect-rich, amorphous CN photocatalyst as well as its characterization (Fig. 1). The investigation of microwave power and irradiation time was conducted to determine their influence on the structure, optical, and surface properties of the achieved graphitic carbon nitride materials. The photocatalytic performance of materials was compared under UV-A, visible, and simulated solar light irradiation using procaine as a model emerging pharmaceutical pollutant. Furthermore, scavenger experiments were performed to identify dominant ROS involved in degradation process. In addition, energy consumption of microwave and conventional thermal synthesis was assessed to highlight the sustainability of this method.

**Table 1:** Overview of previously reported direct microwave synthesis of g-C_3_N_4_

Precursor + MW absorber	Synthesis conditions	Use	Ref.
Cyanuric chloride + sodium azide	350 W up to 180 °C, hold 30 min	Synthesis	21
Urea + CuO	1 kW, 6–18 min	H_2_ evolution	22
Melamine + artificial graphite powder	4 kW up to 560 °C, 10 min	Dye degradation	23
Melamine + carbon fibers	∼700 W, 2–5 min	Field emission	24
Thiourea + CuO	∼700 W, 15–30 min	N_2_ photofixation	25
Urea + MW absorbing crucible	750 W, 11–16 min	Dye degradation	26
Melamine + cyanuric acid	700 W 20–40 min	Dye degradation	27
Melamine + carbon fibers	4.5 kW up to 560 °C, hold 10 min	Drug carrier	28
Urea + MW absorbing kiln layer	600–800 W, 9–15 min	Dye degradation	29
Urea + n.a.	800 W up to 170 °C, hold 15 min	Dye degradation, H_2_ evolution	30

Overall, the core novelty and advantage of this study is in synthesis of a CN material using an unmodified, standard microwave oven and conventional ceramic crucibles, with the addition of silicon carbide (SiC) susceptors. Unlike bulk additives, SiC susceptors are physically isolated from the reaction phase, they deliver rapid heating profiles and can be easily separated from the final product without the risk of contamination or process interference. Furthermore, without requiring professional laboratory equipment or pre-step treatments, a well-defined CN framework was successfully synthesized even at a low microwave power of 600 W, demonstrating a highly possible pathway for sustainable production of photocatalysts.

## Experimental

### Materials and chemicals

All reagents were of analytical grade and used without further purification. Urea (99.5%, VWR International, USA) was used as a precursor for the synthesis of graphitic carbon nitride. Procaine (Thermo Fisher Scientific, USA) was selected as a model pharmaceutical pollutant. Isopropanol (IPA, Gram Mol, Croatia), formic acid (FA, Lach-Ner s.r.o., Czech Republic), *p*-benzoquinone (BQ, Merck KGaA, Germany), and sodium azide (SA, Kemika, Croatia) were used as scavengers for photocatalytic mechanism studies. All experiments were conducted in ultrapure water (18.2 MΩ cm).

### CN synthesis

Graphitic carbon nitride photocatalysts were synthesized *via* rapid microwave polymerization using urea as a precursor. In a typical procedure, 5 g of urea was added to a covered mid-high ceramic crucible together with two cylindrical (*Ø* 10 mm) microwave-absorbing silicon carbide (SiC) susceptors, which were used to enhance microwave absorption and promote uniform heating. The crucible was then placed at the center of the rotating glass plate in a microwave reactor (Samsung ME73A, South Korea) operating at 2.45 GHz.

A total of six CN samples were prepared under different microwave irradiation conditions. Three samples were synthesized at a magneton power of 600 W for 10, 12, and 15 min, while the additional three samples were obtained at 800 W for the same irradiation times. Immediately following the synthesis, the temperature inside the ceramic crucible was determined to exceed 500 °C, as measured by K-type thermocouple connected to a Fluke 724 temperature calibrator with automatic cold-junction compensation. This indicates that the peak temperatures generated within the microwave during the reaction were even higher. The resulting materials were denoted as CN*x-y*, where *x* represents the applied microwave power (600 and 800 W), while *y* represents synthesis time (10–15 min). The ceramic crucible was immediately removed from the microwave post-synthesis, and the obtained yellow powders were rapidly cooled to room temperature and ground prior to further characterization and photocatalytic evaluation. For comparison, a reference CN sample was prepared by conventional thermal polymerization in an electrical furnace (Inko LP-08, Croatia), following a previously reported procedure.^31,32^ Briefly, 30 g of urea was placed in a ceramic bowl covered with aluminum foil and heated at 550 °C for 2 h with a heating rate of 3 °C min^−1^. The resulting powder was labeled as CN550.

### Characterization of photocatalyst

Analytical and spectroscopic techniques used to understand the structure, surface chemistry, morphology, and optical properties of the obtained graphitic carbon nitride powders include FTIR, XRD, XPS, DRS, SEM, and nitrogen physisorption measurements.

Fourier-transformation infrared (FTIR) spectra were recorded on a Shimadzu IRSpirit (Japan) spectrometer equipped with an attenuated total reflectance (ATR) attachment.

X-ray diffraction (XRD) patterns of the powder samples were collected using a Bruker D8 Advance diffractometer (USA) operating in the Bragg–Brentano configuration with Cu Kα radiation (8.04 keV). Data were acquired with a step size of 0.02° in 2*θ* and a retention time of 1 s.

Diffuse reflectance spectroscopy (DRS) was performed on a PerkinElmer Lambda 1050+ spectrophotometer (USA) equipped with an integrating InGaAs sphere. The optical band gap energies (*E*_g_) were estimated from Kubelka–Munk plots derived from the reflectance spectra.

X-ray photoelectron spectroscopy (XPS) measurements were carried out on a SPECS instrument under ultra-high vacuum (UHV) conditions, using a monochromated Al Kα X-ray source (*ℏν* = 1486.74 eV) and a Phoibos MCD 100 electron energy analyzer. Survey spectra were recorded at a pass energy of 50 eV, while high-resolution spectra were measured at 10 eV. Bandgap energy (*E*_g_) was determined from the slopes of the Tauc plots obtained from the data.

Scanning electron microscopy (SEM) imaging was conducted using a Tescan Vega III Easyprobe microscope (Czech Republic) operated at 10 kV and equipped with a secondary (SE) and backscattered electron (BSE) detectors.

Transmission electron microscopy (TEM) characterization was performed on a JEOL JEM-1400Flash microscope at an operating voltage of 120 kV.

Nitrogen adsorption–desorption isotherms were measured at −196 °C using a Micromeritics ASAP 2000 instrument (Norcross, USA). The specific surface area (*S*_BET_) was calculated using the Brunauer–Emmett–Teller (BET) method within the relative pressure range (*p*/*p*_0_) of 0.0–0.2. The pore size distribution was determined from the nitrogen adsorption branch using the Barrett–Joyner–Halenda (BJH) model.

### Photocatalytic activity evaluation

The photocatalytic performance of the prepared CN*x-y* materials was evaluated through the degradation of procaine (PRO) in aqueous solution. A PRO solution was prepared at a concentration of 5 mg L^−1^. Firstly, 25 mg of photocatalyst was dispersed in 100 mL of PRO solution in a cylindrical glass reactor. Prior to irradiation, the suspension was magnetically stirred in the dark for 30 min to establish adsorption–desorption equilibrium.

Photocatalytic degradation studies were carried out using three different irradiation sources: UV-A light (70 W, *λ* = 365 nm), visible light (100 W, *λ* = 450 and 600 nm), and simulated solar light (SSL, 430 W). During irradiation, 5 mL samples were withdrawn at regular time intervals (0, 5, 10, 20, 30, 45, and 60 min) for UV-A and SSL experiments, while additional sampling times at 90 and 120 min were included for visible light tests. The collected samples were immediately filtered through 0.45 µm mixed cellulose ester (MCE) membranes to remove photocatalyst particles. The residual PRO concentration was determined by UV-Vis spectroscopy (Hewlett-Packard HP 8340, USA) by monitoring the characteristic absorption maximum at 290 nm. Additionally, the selected photocatalyst was evaluated by HPLC analysis to confirm the degradation of procaine and reusability. Photolysis experiments were performed in the absence of a photocatalyst to confirm that the degradation of PRO under light irradiation was negligible. All experiments were conducted at 25 °C using a thermostatic bath to maintain constant temperature. The efficiency of microwave-synthesized CN*x-y* samples was compared to the conventionally prepared reference sample CN550. The degradation efficiency was calculated according to eqn (1):1
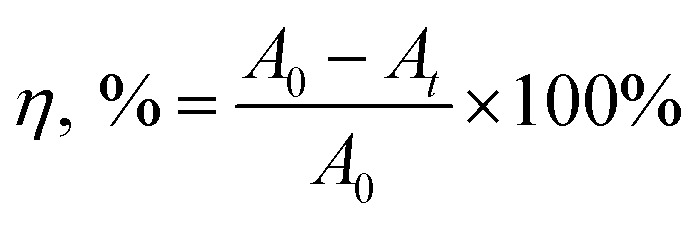
where *A*_0_ is the initial absorbance of PRO and *A*_*t*_ is the absorbance at irradiation time *t*. The photodegradation kinetics of the PRO were calculated by the pseudo-first-order (PFO) model:2
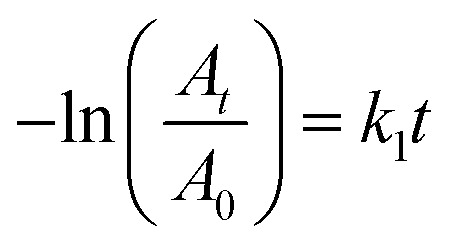
where *k*_1_ is the reaction rate constant.

### Reactive mechanisms experiments

To determine photocatalytic mechanisms and identify the dominant reactive oxygen species (ROS), scavenger experiments were performed under solar irradiation using the most active photocatalyst, CN800-15. Specific scavengers were introduced into PRO solution to selectively quench oxidative species and charge carriers: isopropanol (IPA, ˙OH^−^ scavenger), formic acid (FA, hole scavenger), *p*-benzoquinone (BQ, ˙O_2_^−^ scavenger), and sodium azide (SA, ^1^O_2_ scavenger). The suspension with added scavengers was irradiated under SSL, following the same procedure as photocatalytic experiments. PRO degradation was quantified using high-performance liquid chromatography (HPLC, Agilent 1100 HPLC-DAD, USA). The separation of procaine was achieved on an XBridge C18 column (150 mm × 4.6 mm, 3.5 µm) using gradient elution with 0.1% formic acid in water and acetonitrile at a flow rate of 0.5 mL min^−1^. PRO was detected at 290 nm with a retention time of approx. 8.1 min.

### Energy consumption estimation

The electrical energy demand of both synthesis methods was measured using an EMOS P5822 digital power meter (Czech Republic). The values were measured per batch and normalized per gram of obtained photocatalyst after synthesis. This analysis evaluated the sustainability of the microwave process compared to conventional thermal polymerization.

## Results and discussion

### Characterization of photocatalysts

**Fig. 2:**
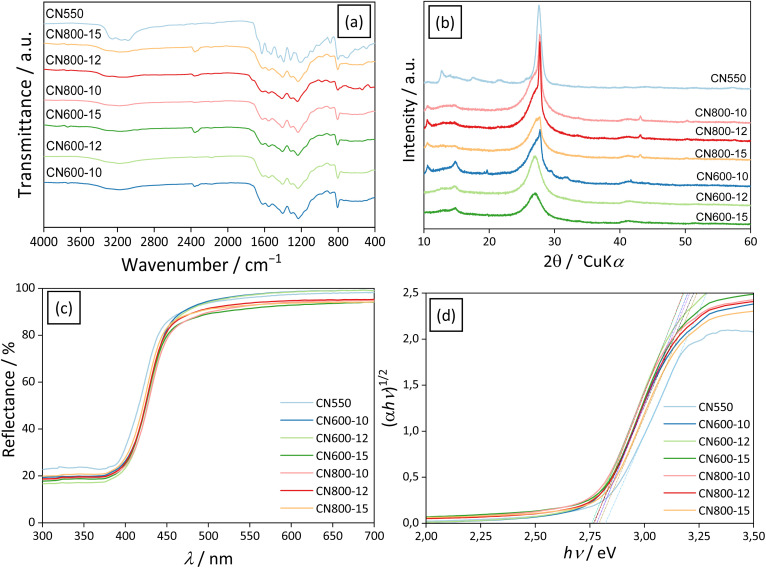
Structural and optical characterization of microwave CN samples and conventionally synthesized CN550: (a) FTIR spectra, (b) XRD patterns, (c) UV-Vis diffuse reflectance spectra, and (d) corresponding Tauc plots.

**Fig. 3:**
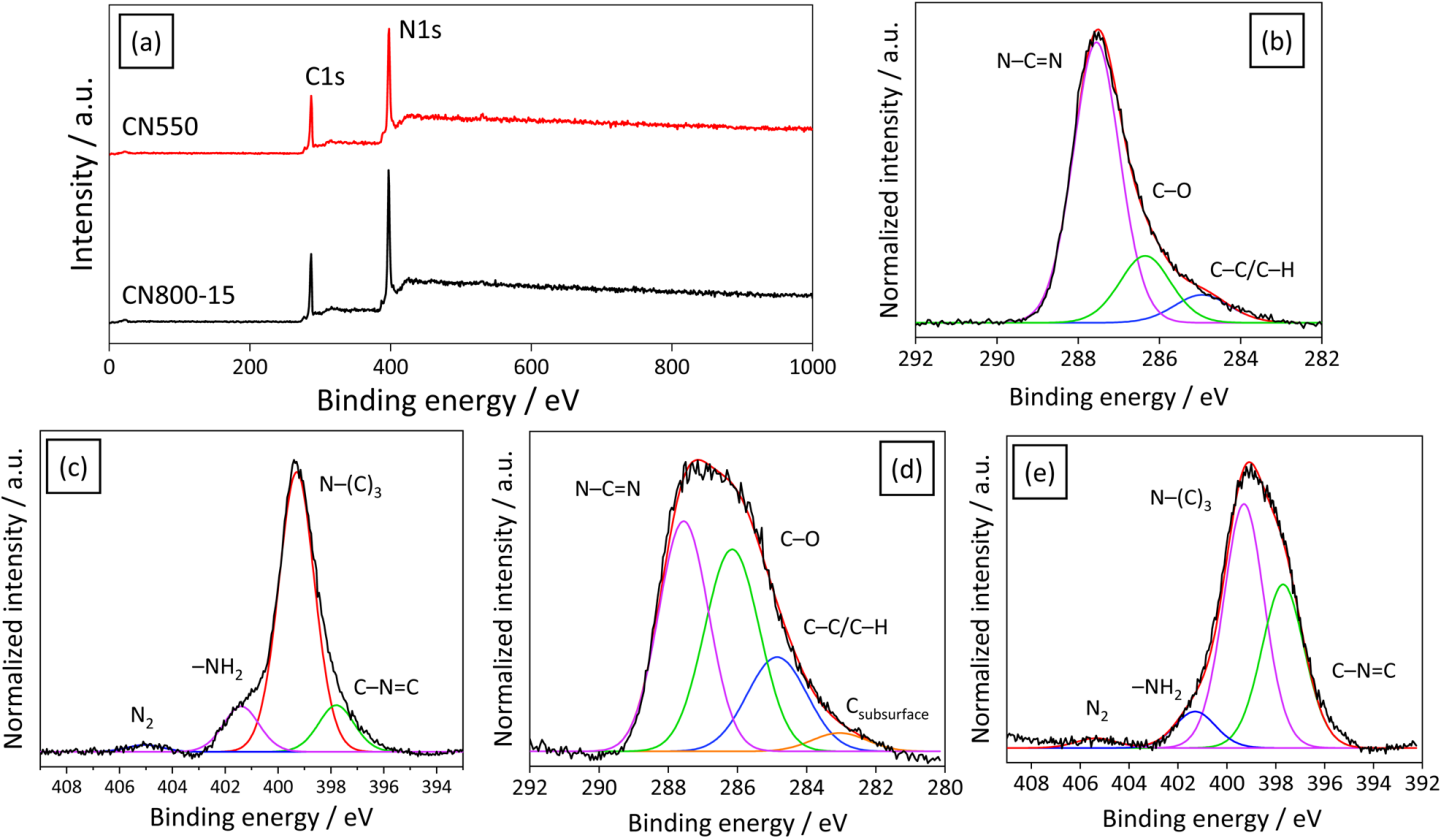
XPS characterization of CN550 and CN800-15 samples: (a) survey spectra, (b) C 1s spectra of CN550, (c) N 1s spectra of CN550, (d) C 1s spectra of CN800-15, and (e) N 1s spectra of CN800-15.

FTIR spectra (Fig. 2a) show that all materials exhibit characteristic vibrational features of graphitic carbon nitride, confirming the successful CN formation. The band located at ∼800–880 cm^−1^ is attributed to the bending vibration of the heptazine (*s*-triazine) ring units, while the series of bands in the 1200–1700 cm^−1^ region corresponds to stretching vibrations of C–N and C=N rings. In particular, the peaks at 1240 and 1320 cm^−1^ can be attributed to C–NC–C and C–NH–C stretching vibrations associated with the condensed CN network. The broad feature observed between 3100 and 3300 cm^−1^ is associated with N–H stretching vibrations of residual amino groups. Additionally, the signal at 3350 cm^−1^ is typically related to the O–H functional group due to adsorbed water.^33–35^ Compared to CN550 sample, MW samples show broader and less-defined aromatic stretching bands in the 1200–1700 cm^−1^ region and a broader contribution in the 3000–3400 cm^−1^ region (–NH_2_/–NH groups and adsorbed hydroxyl species).^36^ Those differences suggest that MW synthesis affects the polymerization pathway, resulting in variations in the degree of functional groups and structural defects. The sharper, more resolved fingerprint of CN550 indicates a more ordered and complete condensation compared to MW samples. These variations in the degree of polymerization are consistent with existing literature suggesting that microwave-assisted protocols, while efficient, often result in materials with poorer crystallinity and higher densities of defect sites compared to conventional pyrolysis.^37^ The XRD patterns (Fig. 2b) further confirm the formation of graphitic carbon nitride. Two characteristic diffraction features can be observed for all samples: 13.1° assigned to the in-plane stacking of heptazine units in (100) plane, and the intense peak at 27.4°, corresponding to the interlayer stacking of aromatic layers in (002) plane (ICDD PDF # 00-87-1526).^29^ Notably, the CN*x-y* samples exhibit broader diffraction peaks and reduced intensity of the (100) plane compared to CN550, indicating decreased in-plane order and lower crystallinity. In addition, a slight 2*θ* shift suggests changes in interlayer stacking and structural distortion induced by rapid microwave heating, supporting the formation of a more disordered, defect-rich CN structure.^38,39^ The DSR spectra (Fig. 2c) of all samples display a characteristic absorption edge in the visible region (∼420–450 nm), confirming the nature of the g-C_3_N_4_ semiconductor.^40^ Compared to CN550, microwave-synthesized samples exhibit a slight redshift, particularly at higher microwave power and longer irradiation time. The characterization is in agreement with the enhanced visible-light absorption of procaine for the microwave samples, as well as the slight narrowing in the optical band gap (Fig. 2d). The microwave-prepared samples exhibit band gap energies in the range of 2.68–2.72 eV, whereas CN550 shows a slightly larger band gap of 2.88 eV (Table 2). A slight increase in the band gap energy of microwave samples with the increase of the synthesis time could be attributed to the more structurally ordered and crystallized CN network.^41,42^ The reduced band gap suggests improved visible-light absorption, which contributes to the enhanced photocatalytic activity under visible irradiation.^35^ This increased absorption, coupled with the observed structural disorder, suggests that the microwave-assisted route effectively tunes the electronic band structure by modulating the condensation degree. The survey spectra (Fig. 3a) confirm the presence of C and N as the dominant elements in both microwave- and furnace-synthesized materials. The high-resolution C 1s spectra in Fig. 3b display a dominant peak at 288.0–288.4 eV, assigned to sp^2^-hybridized carbon in N–C=N coordination, originating from the heptazine units. This peak is recognized as a fingerprint of the polymerized g-C_3_N_4_ structure. Similarly, around 286.0–286.5 eV, C–O species occur typically due to surface oxidation, hydroxyl/ether groups, or absorbed oxygen species. At the binding energy of 284.6–285.0 eV, C–C/C–H bonds are visible, generally arising from adventitious carbon contamination or defect-related carbon species.^43^ Additionally, in CN800-15, *C*_subsurface_ is observed between 282.0 and 283.0 eV (Fig. 3d), indicative of structural defects or carbon-rich domains. Compared to CN550, the broader C 1s with stronger C–O and C–C/C–H contributions in CN800-15 suggest a higher defect density and slightly reduced polymerization due to rapid microwave heating. Such defects and oxygen-containing functional groups may act as electron or hole trapping centers, thereby explaining the superior degradation rate despite the reduced crystallinity.^44,45^ Nevertheless, excessive defect density may also serve as recombination centers, indicating that an optimal balance between structural order and defect concentration is crucial. The high-resolution N 1s spectra for both materials (Fig. 3c and e) can be deconvoluted into characteristic peaks at 398.6 eV, 400.0 eV, 401.0–401.4 eV, and 404.0–405.0 eV, corresponding to sp^2^-hybridized nitrogen in C configuration, tertiary nitrogen N–(C)_3_, (–NH_2_) amino groups, and N_2_-related species, respectively.^46^ Notably, the CN800-15 sample has a higher ratio of sp^2^-hybridized nitrogen to tertiary nitrogen compared to CN550, suggesting that the rapid, localized heating in the microwave process promotes different polymerization kinetics, leading to a more pronounced heptazine-based framework with altered defect density.^21^ To evaluate the structural stoichiometry induced by different synthesis methods, the atomic C/N ratios of the CN550 and CN800-15 samples were calculated from the high-resolution C 1s and N 1s spectra. The CN550 sample exhibited an atomic C/N ratio of 0.754. This value closely aligns with the ideal theoretical ratio of pristine g-C_3_N_4_ (0.750),^47^ confirming that prolonged thermal polymerization successfully yields a stoichiometric heptazine network. CN800-15 sample, however, displayed a noticeably higher C/N ratio of 0.869, shifting the material toward a carbon-rich (and nitrogen-deficient) structure.^48^ These results provide deep insights into the defect-formation mechanism characteristic of microwave synthesis. Unlike slow equilibrium heating of an electric furnace, microwave radiation causes ultra-fast, volumetric heating through molecular dipole rotation. At high power (800 W), local hot spots and high thermal kinetic energy are generated in the precursor. This rapid energy dissipation affects less thermally stable amino groups (–NH_2_) and nitrogen atoms in triazine/heptazine rings.

**Table 2:** Summary of band gap energies, BET surface area, and pore size for all samples

CN sample	Band gap energy	Specific surface area	Pore size
	*E*_g_, eV	*S*_BET_, m^2^ g^−1^	*d*_avg_, nm
600–10	2.68	33.9	6.4
600–12	2.71	24.3	7.0
600–15	2.71	42.9	7.3
800–10	2.68	37.8	9.4
800–12	2.71	34.5	8.1
800–15	2.72	39.8	5.1
CN550	2.88	74.2	13.0

**Fig. 4:**
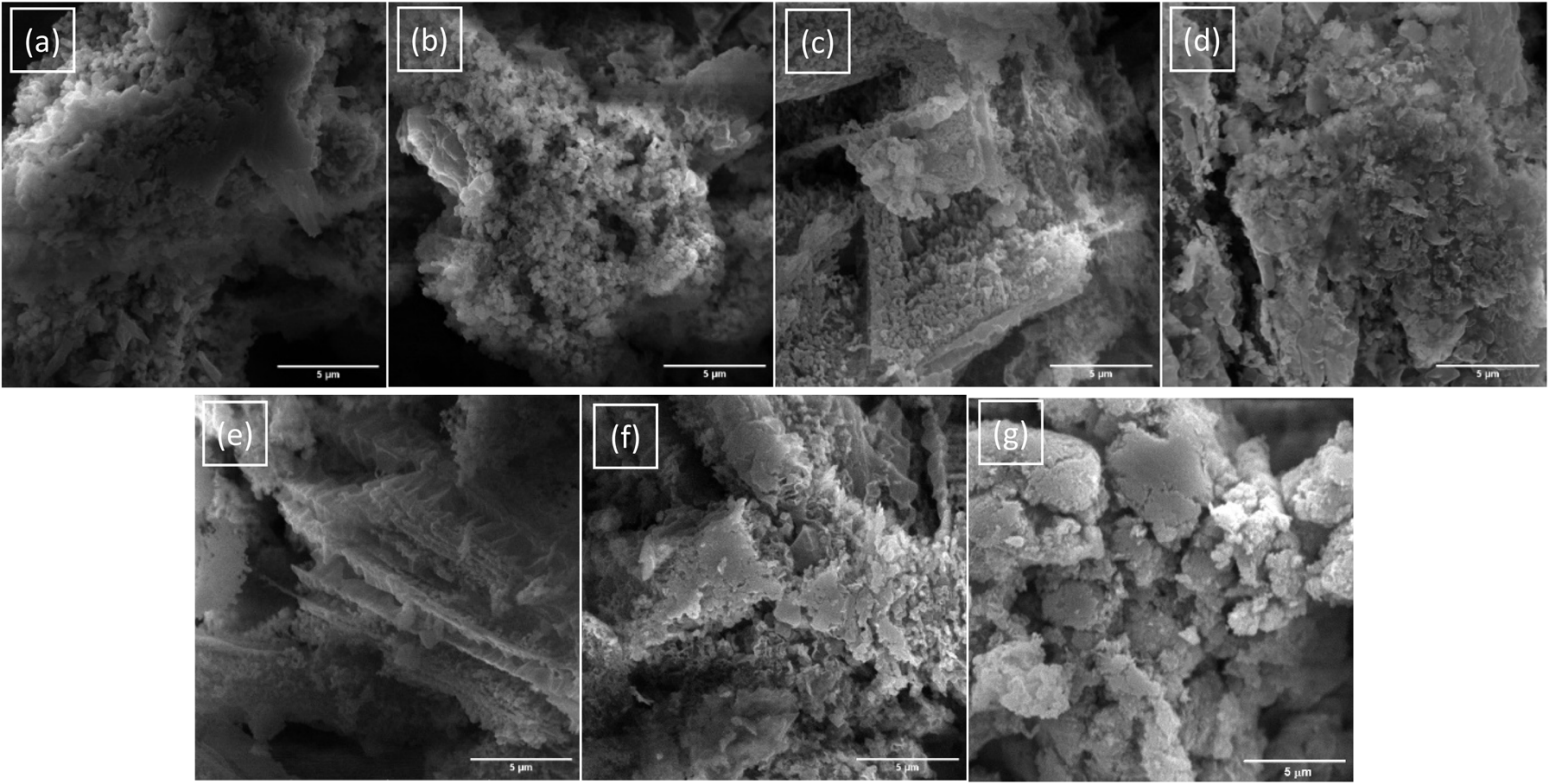
SEM micrographs of (a) CN600-10, (b) CN600-12, (c) CN600-15, (d) CN800-10, (e) CN800-12, (f) CN800-15 samples, and (g) CN550 sample.

As these nitrogen species volatilize as gaseous compounds (*e.g.* NH_3_), structural vacancies (defects) after rapid cooling to ambient temperature are intentionally left behind in the framework instead of being healed through long-term thermal annealing. This nitrogen depletion increases the relative proportion of structural carbon (N–C=N), explaining the higher C/N ratio of 0.869. Such introduction of highly advantageous defects in CN structure, acts as electron traps, changes electronic band structures, and improves the separation of generated electrons and holes, which directly leads to a higher degradation efficiency.^45^ According to IUPAC classification, the obtained nitrogen adsorption–desorption isotherms can be classified as type III, characteristic of nonporous or macroporous solids (S1, S2).^49^ A minor hysteresis loop is observed, although it is not particularly pronounced and does not correspond to any specific IUPAC hysteresis subtype. Overall, the isotherms indicate predominantly non-microporous structures with reversible adsorption characteristics. Furthermore, the majority of pores fall into the mesoporous (2–50 nm) and macroporous (>50 nm) range (Table 2). The CN*x-y* samples exhibit average pore diameters between 5.1 and 9.4 nm, while CN550 sample shows slightly larger pore sizes with a higher specific surface area.^50^ The higher surface area of CN550 can be attributed to a more developed porous structure with a larger contribution from macropores (∼100 nm) compared to CN*x-y*. No substantial differences were observed among CN*x-y* samples as a function of time and microwave power, indicating that microwave synthesis primarily affects structural disorder and defect formation rather than porosity. SEM micrographs presented in SEM micrographs in Fig. 4 show relatively similar morphology for all synthesized samples. The only observable difference lies in the quantity of smaller particles formed on the surfaces of the larger crystals when comparing the microwave-synthesized samples at 600 and 800 W. Samples synthesized at 600 W (Fig. 4a–c) show a significantly rougher morphology than those synthesized at 800 W (Fig. 4d–f). Moreover, these observations correlate relatively well with the BET and XRD results, suggesting that most of the structural changes occur at meso- and microscale. Thus, the enhanced photocatalytic efficiency of CN800-15 cannot solely be attributed to textural properties, but rather to the defect-rich structure generated by rapid microwave synthesis.^26^

**Fig. 5:**
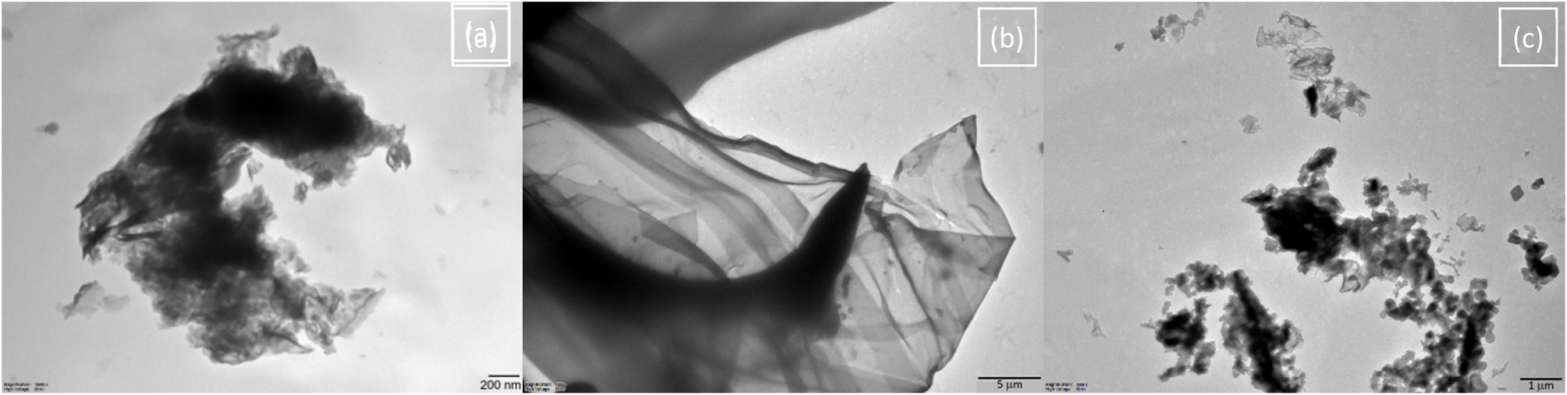
TEM images of (a) CN550 and (b and c) CN880-15 sample.

To gain a deeper insight into their structural transitions at the nanoscale, TEM analysis was conducted on samples CN550 and CN800-15 (Fig. 5). The conventionally synthesized sample CN550 (Fig. 5a) exhibits a characteristic dense, compact, and multi-layered morphology with well-defined and smooth boundaries. This formation reflects a slow thermal polycondensation in the furnace, which promotes layer stacking with minimal structural strain. In stark contrast, the microwave synthesis triggers a radical morphological transformation. The optimized matrix transitioned into ultra-thin, highly transparent and wrinkled nanosheet (Fig. 5b). This silk-like surface is a direct consequence of the rapid, volumetric dielectric heating – the explosive release of volatile gaseous byproducts (NH_3_ and CO_2_) thermally exfoliates and etches the bulk structure from within. Furthermore, the high-power microwave exposure promotes the development of a highly open, sponge-like network with a high density of interconnected holes (Fig. 5c), which structurally justifies the available surface active sites. Crucially, the surface corrugation and torn edges visible across the microwave nanosheets serve as a footprint of lattice distortion induced by trapped nitrogen vacancies. This structure shortens the migration distance of photogenerated charge carriers to the photocatalyst surface, and in addition to shallow defect traps, suppresses charge recombination and maximizes procaine degradation rate.

### Photocatalytic activity

**Fig. 6:**
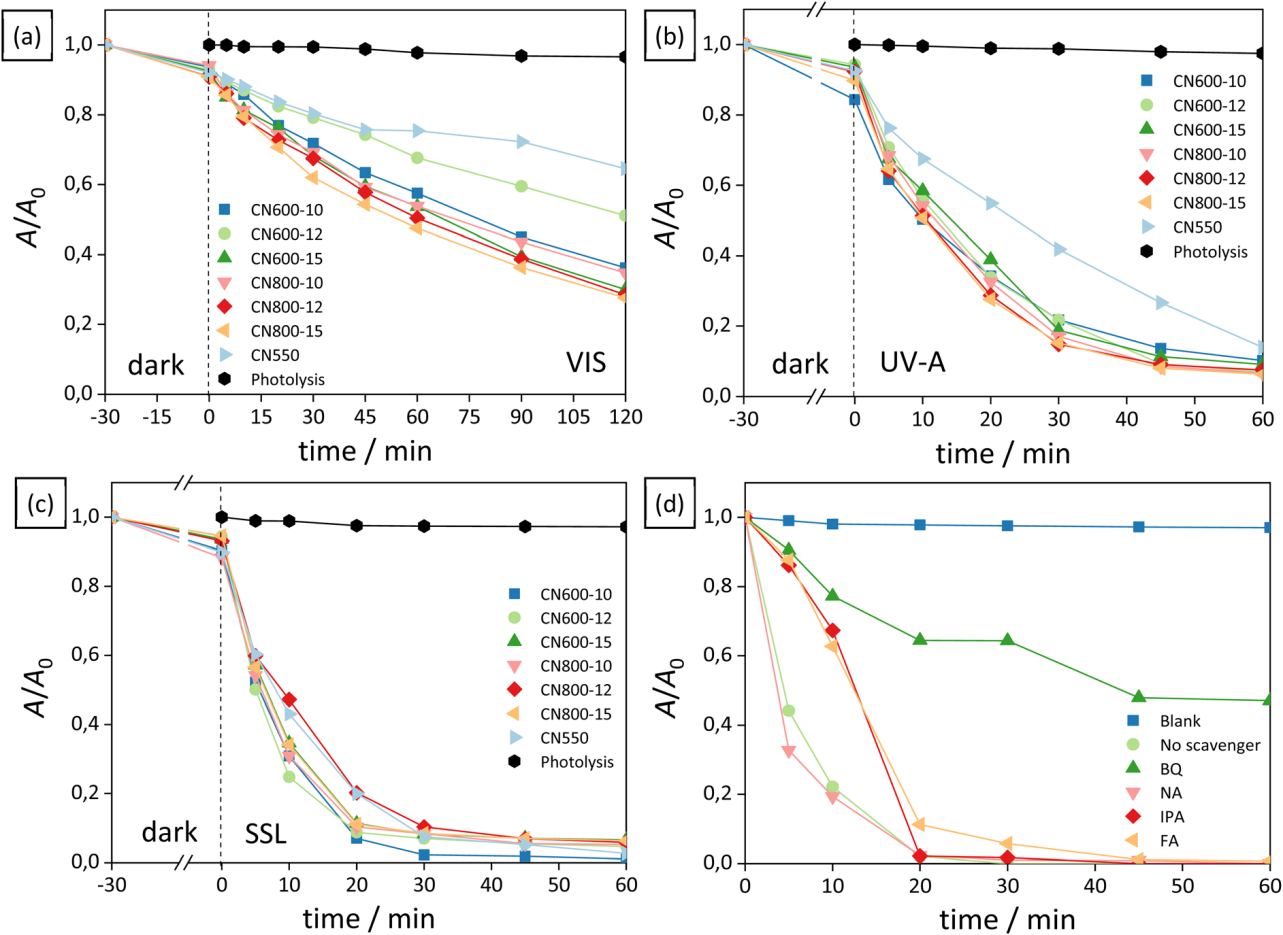
Photocatalytic degradation of procaine under (a) visible light, (b) UV-A light, (c) simulated solar light, and (d) scavenger studies on photocatalytic degradation of procaine using CN800-15 sample under simulated solar light.

The photocatalytic activity of the synthesized graphitic carbon nitride materials was evaluated through the degradation of pharmaceutical procaine (PRO) under UV-A irradiation, visible light (VIS), and simulated solar light (SSL). Fig. 6 presents the results of the photocatalytic degradation and corresponding efficiencies of all prepared photocatalysts under three irradiation sources. Under visible light irradiation (Fig. 6a), the microwave-synthesized CN800-15 sample showed the highest activity, achieving 72.43% PRO degradation within 120 min, which is more than double that of the conventionally prepared CN550 material (35.42%). Importantly, all microwave-synthesized samples outperformed CN550 under visible light, confirming the beneficial role of microwave-induced structural defects and structural disorder.

Similarly, under UV-A irradiation (Fig. 6b), CN800-15 reached 93.75% degradation after 60 min, compared to 86.08% for CN550. In contrast, under simulated solar light (Fig. 6c), both microwave and conventionally synthesized samples showed high degradation efficiencies (>90%) within 60 min. The CN550 sample exhibited slightly higher degradation efficiency (97.37%) compared to CN800-15 (93.73%), suggesting that successful degradation is influenced by the combination of UV-A and visible light irradiation. Due to the low PRO adsorption in the dark (<10%), adsorption kinetics were not determined as they did not play a dominant factor in the photocatalytic process. From the obtained results, it can be deduced that under SSL and UV-A light, the photocatalytic degradation of procaine proceeded rapidly due to the high-energy flux, thus achieving almost complete degradation in 60 min. In contrast, visible light has lower photon energy, so the reaction kinetics were much slower, requiring a longer irradiation time (120 min). In brief, different behaviors under each light source are as follows: (a) VIS: synergistic effect of narrow bandgap and nitrogen defects that act as electron traps; (b) UV-A: high-energy photons easily excite electrons in all materials, leading to fast degradation. CN*x-y* samples perform better due to defect charge separation, and (c) SSL: combination of both visible and UV light leads to competition between near-pristine CN550 material and CN*x-y*, while CN550 works better under UV-A light, CN*x-y* shows its potential under VIS light, so their efficiencies basically level out. As summarized in Table 3, the reaction rate constants confirm the significantly enhanced kinetics of the microwave-synthesized samples. Under VIS light, *k* of CN800-15 was 3.4 times and 2.8 times higher under UV-A light than that of CN550. Under SSL irradiation, CN550 showed only a slightly higher degradation rate (≈1.2×) compared to CN800-15, which is consistent with the observed differences.

**Table 3:** Pseudo-first-order rate constants (*k*_1_) and half-life times (*t*_1/2_) of PRO degradation under different radiation sources using CN samples

CN sample	VIS		UV-A		SSL	
	*k*_1_, min^−1^	*t*_1/2_, min	*k*_1_, min^−1^	*t*_1/2_, min	*k*_1_, min^−1^	*t*_1/2_, min
CN600-10	0.0085	81.5467	0.0382	18.1452	0.0899	7.7102
CN600-12	0.0046	150.6842	0.0504	13.7529	0.0534	12.9803
CN600-15	0.0090	77.0164	0.0471	14.7165	0.0530	13.0782
CN800-10	0.0091	76.1700	0.0531	13.0536	0.0531	13.0536
CN800-12	0.0095	72.9629	0.0510	13.5911	0.0571	12.1392
CN800-15	0.0114	60.8024	0.0534	12.9803	0.0526	13.1777
CN550	0.0034	203.8668	0.0189	36.6745	0.0613	11.3075

### Scavenger testing

Fig. 6d presents the photocatalytic degradation of PRO *via* CN800-15 sample in the presence of selective scavenger agents under SSL, providing insights into dominant reactive species in degradation process. Four scavengers were used: isopropanol as a hydroxyl radical (˙OH) scavenger, formic acid as a hole (h^+^) quencher, *p*-benzoquinone as a superoxide anion (˙O_2_^−^) scavenger, and sodium azide as a singlet oxygen (^1^O_2_) trapping agent.^51–53^

From the observed diagram, it is evident that the addition of *p*-benzoquinone has a considerable impact on the PRO degradation rate, indicating that superoxide anions (˙O_2_^−^) represent the primary reactive oxygen species in the photocatalytic process.^31^ In the presence of BQ, the kinetic rate constant decreased to 0.0127 min^−1^, resulting in approximately 15× increase in the degradation half-life (*t*_1/2_ = 54.58 min), compared to the experiment where no scavengers were added (Table 4). Formic acid also caused a noticeable reduction in PRO removal, suggesting that the photogenerated holes (h^+^) have a minor role in the degradation process. From the latter, it can be concluded that photogenerated electrons (e^−^) react with dissolved O_2_ and reduce it to ˙O_2_^−^, which participate in the oxidative degradation of PRO molecules.^54^ Due to the relatively positive valence band position of graphitic carbon nitride (∼1.4 V *vs.* NHE), the direct oxidation of H_2_O or OH^−^ into hydroxyl radicals is thermodynamically less favorable. This explains the limited impact of ˙OH on degradation rate, as it can be seen in the literature.^55,56^ Nitrogen vacancies generated beneath the conduction band are strictly seen as shallow electron traps rather than deeper recombination centers. Therefore, instead of “firmly” holding the electrons, they simply spatially segregate them from the photogenerated holes in the valence band, suppressing the recombination. As confirmed by TEM analysis, microwave synthesis yields ultrathin nanosheet material.

**Table 4:** Pseudo-first-order constants and half-life times of PRO degradation using CN800-15 sample under SSL

Scavenger	*k*_1_, min^−1^	*t*_1/2_, min
Isopropanol	0.1955	3.71
Formic acid	0.1104	7.58
*p*-benzoquinone	0.0127	54.58
Sodium azide	0.1866	6.28
No scavenger	0.0914	3.55

Consequently, these defects are in immediate proximity to the catalyst–water interface, which minimizes diffusion distance of electrons to the surface,^47,57^ and because the conduction band of g-C_3_N_4_ is more negative than the reduction potential of O_2_/˙O_2_^−^, electrons can reduce oxygen into ˙O_2_^−^.^58,60^ There are two possible pathways of procaine degradation: (a) direct decomposition (radicals directly attack the electron-deficient sites of the procaine molecule) or (b) indirect degradation (˙O_2_^−^ can act as a precursor to more aggressive oxidants such as H_2_O_2_ and ˙OH through a series of protonation and reduction steps,^59,61,62^ which subsequently contribute to the mineralization of procaine).

### Photocatalytic stability and reusability

Cycling experiments were performed for PRO degradation using the CN800-15 sample under VIS irradiation. After each run, the photocatalyst was filtered, washed with ultrapure water, dried overnight at 80 °C, and reused in the next degradation cycle.

**Fig. 7:**
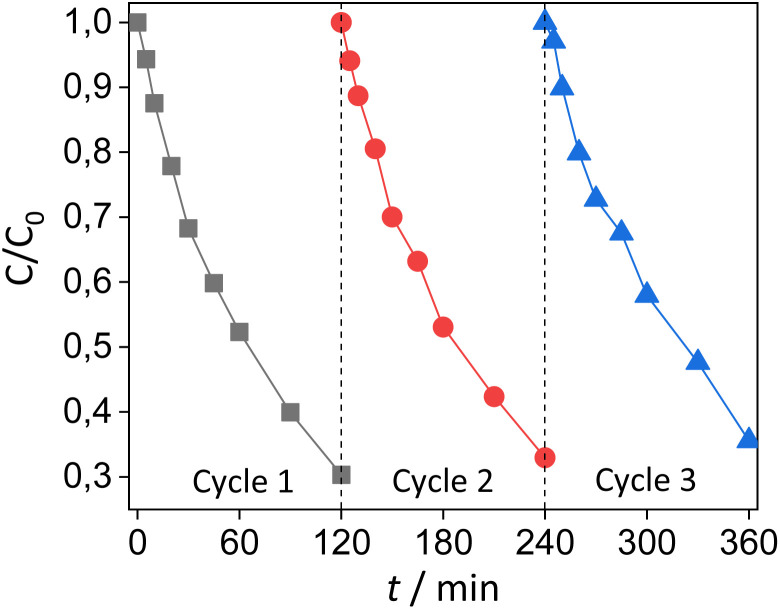
Reusability and stability of CN800-15 photocatalyst during 3 cycles of procaine degradation under VIS light.

As shown in Fig. 7, the degradation efficiency decreased approximately 5.2%, corresponding to 7.5% relative loss after 3 cycles. The relatively high mass loss of photocatalyst particles between cycles (from 25 mg in the first cycle to 15 mg in the last cycle) showed minor influence on photocatalytic degradation, suggesting the possibility of scaling down the mass of used particles in further studies.

### Energy consumption analysis

**Fig. 8:**
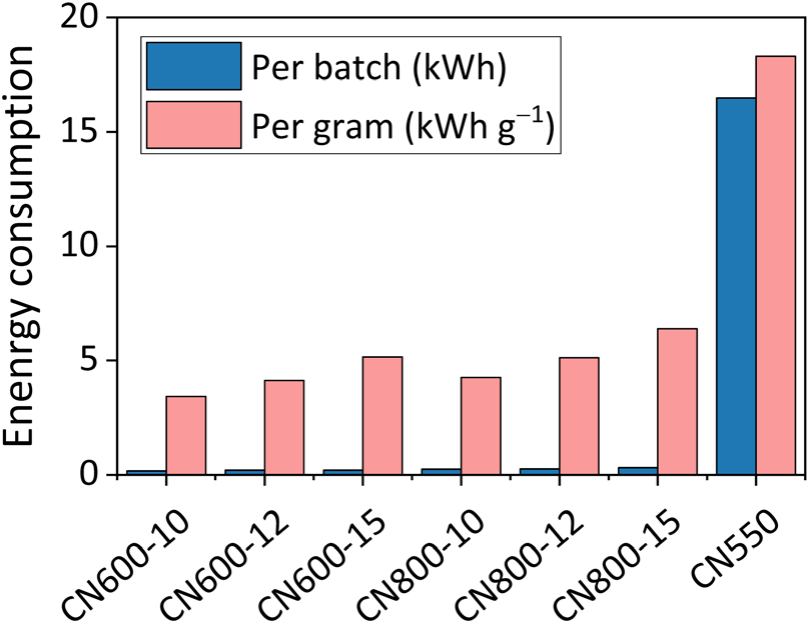
Comparison of energy consumption per batch and per gram of microwave-synthesized samples CN*x-y* and conventionally prepared CN550 in an electrical furnace.

In addition to improved photocatalytic performance, direct microwave synthesis of g-C_3_N_4_ has a substantial advantage in energy saving compared to conventional synthesis in an electrical furnace. Since environmentally sustainable synthesis methods are critical for the real-world implementation of photocatalysts, the electrical energy consumption of both synthesis methods was estimated (Fig. 8). The household microwave synthesis required only short irradiation times (10–15 min), resulting in extremely low energy consumption. The total electrical energy consumption ranged from 0.10 to 0.20 kWh per batch, depending on magnetron power and synthesis duration. Specifically, the CN800-15 sample consumed only 0.20 kWh, with approximately 1% yield, resulting in an energy consumption of 6.4 kWh g^−1^ of photocatalyst. In contrast, conventional synthesis in an electrical furnace involved slow heating (3 °C min^−1^ over ∼3 h) and holding at 550 °C for 2 h, resulting in a process time of 5 h. Under these conditions, the furnace required approximately 16.5 kWh of electrical energy per batch, with approximately 3% yield, equivalent to 18.3 kWh g^−1^ of photocatalyst. Therefore, the conventional synthesis method consumed almost 3× more energy compared to microwave synthesis, thus confirming the rapid and energy-saving characteristic of microwave synthesis.

## Conclusions

In this work, defect-rich graphitic carbon nitride photocatalysts (g-C_3_N_4_) were successfully synthesized *via* an unconventional yet highly efficient direct microwave synthesis in a household microwave oven. Compared with conventional thermal synthesis, this enabled the formation of g-C_3_N_4_ within minutes, providing a rapid and energy-saving alternative for CN production.

Extensive characterization and photocatalytic testing were carried out to determine the structural and functional properties of the samples, as well as their ability to degrade pharmaceutical procaine, under visible (VIS), UV-A, and simulated solar irradiation. FTIR and XRD analysis confirmed the presence of graphitic carbon nitride structure in all CN*x-y* samples. In particular, XPS results revealed that CN800-15 sample possesses a carbon-rich structure with a high amount of nitrogen vacancies (defects), which promote charge separation and ROS generation, thereby leading to superior photocatalytic degradation performance. Moreover, the slightly lower band gap of the CN*x-y* samples (2.68–2.72 eV) improved visible-light activation compared to CN550 (2.88 eV) despite their lower porosity and specific surface area.

The photocatalytic studies using procaine as a model pollutant demonstrated significantly improved degradation rate under visible light. The optimized CN800-15 sample achieved 72.43% degradation within 120 min, outperforming the conventionally synthesized CN550 (35.42%). Radical scavenging experiments identified superoxide anions (˙O_2_^−^) as the dominant species for procaine degradation.

Overall, this study highlights the amazing ability to simply produce defect-rich, amorphous graphitic carbon nitride photocatalyst using only a household microwave. This process consumed ∼3 times less energy per gram of photocatalyst while maintaining high visible-light photocatalytic performance, making it an environmentally and economically viable alternative to conventional thermal synthesis. Lastly, this study offers new findings in microwave synthesis, the possibility of producing photocatalysts used for the removal of various emerging pharmaceuticals, and a significant potential for real-world wastewater treatment applications.

## Author contributions

Debora Briševac: conceptualization, methodology, investigation, formal analysis, data curation, visualization, writing – original draft, writing – review and editing; Floren Radovanović-Perić: methodology, investigation, formal analysis, writing – review and editing; Kristina Tolić Čop: investigation, writing – review and editing; Gordana Matijašić: investigation, writing – review and editing; Lidija Ćurković: supervision, writing – review and editing; Davor Ljubas: conceptualization, supervision, writing – review and editing.

## Conflicts of interest

The authors declare that they have no known competing financial interests or personal relationships that could have influenced the work reported in this paper.

## Supplementary Material

RA-016-D6RA03829H-s001

## Data Availability

The data supporting this article are included as part of the supplementary information (SI). Supplementary information: pore distribution curves and nitrogen adsorption–desorption isotherms. See DOI: https://doi.org/10.1039/d6ra03829h.
