# Supplementary material for: Ultrafast microwave synthesis of defect-rich graphitic carbon nitride for enhanced photocatalytic degradation of procaine
Source: RSC Adv. 2026 Jul 3;16(35):36033–44. doi: 10.1039/d6ra03829h (PMC13330177; doi:10.1039/d6ra03829h)
Supplement: RA-016-D6RA03829H-s001 [file RA-016-D6RA03829H-s001.pdf]

Supplementary Information (SI) for RSC Advances.
This journal is © The Royal Society of Chemistry 2026

SUPPLEMENTARY INFORMATION

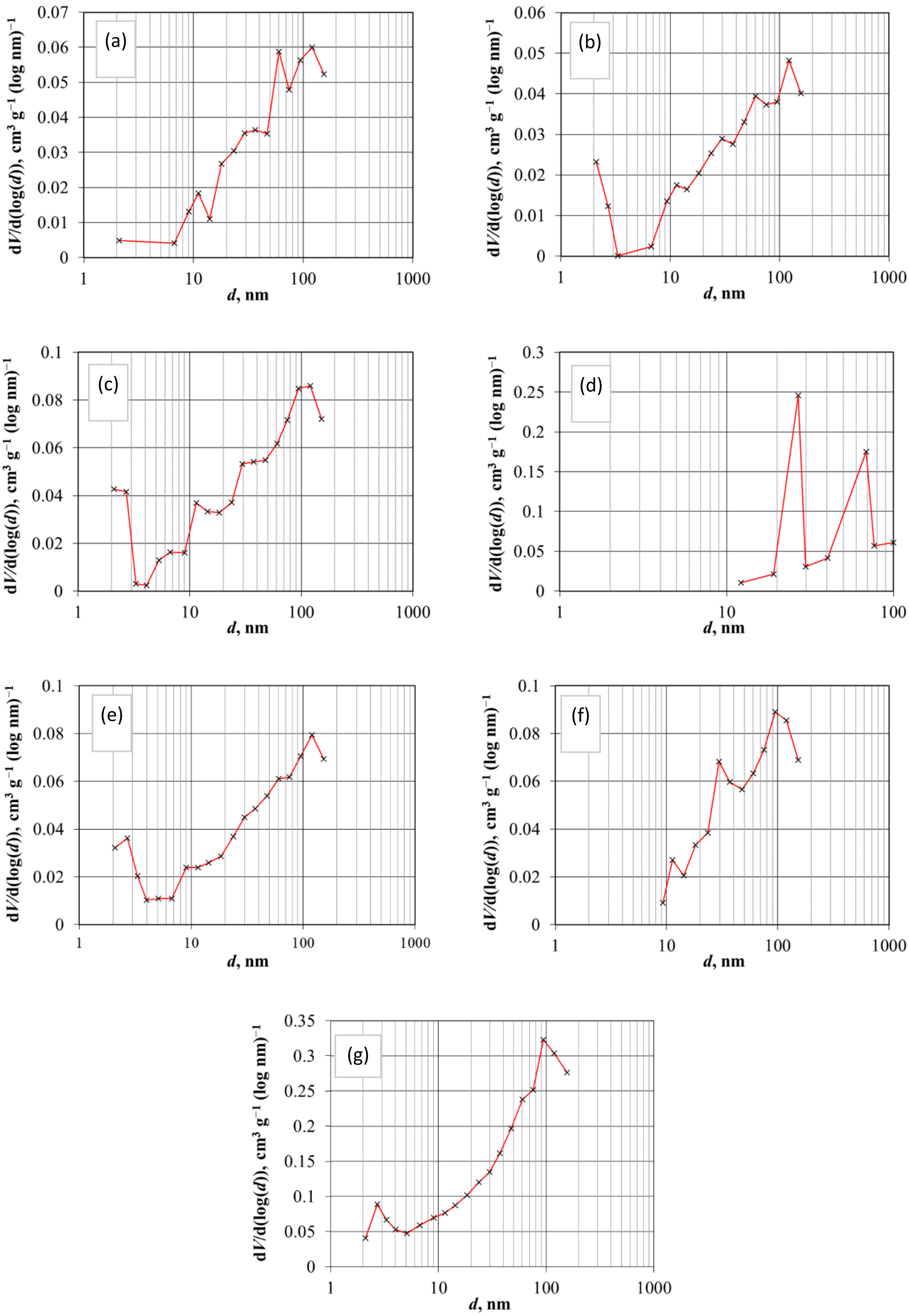

Fig. S1 Pore distribution curves.of prepared CN samples (a) CN600-10, (b) CN600-12, (c) CN600-15, (d) CN800-10, (e) CN800-12, (f) CN800-15, and (g) CN550.

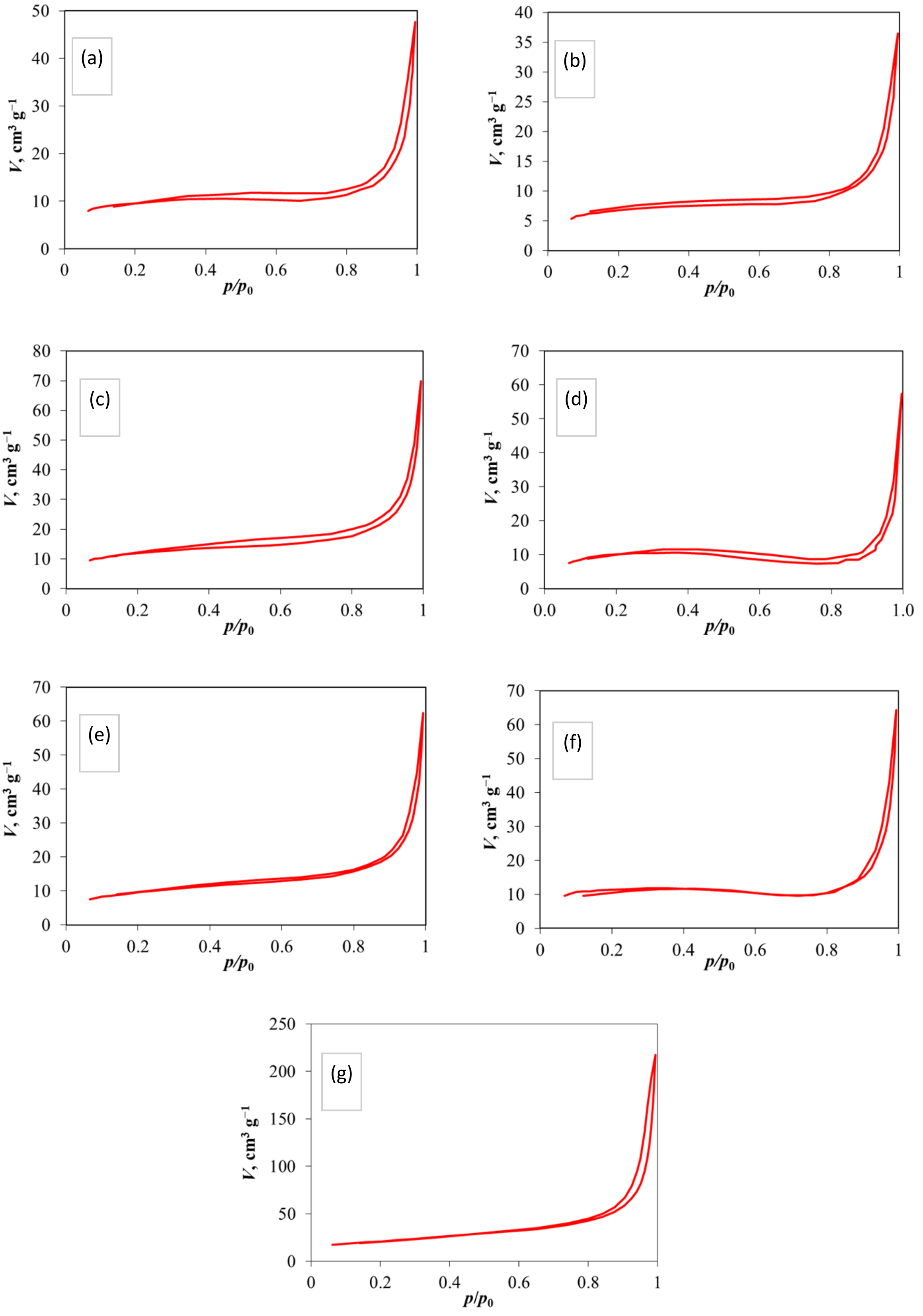

Fig. S2 Nitrogen adsorption-desorption isotherms for CN samples (a) CN600-10, (b) CN600-12, (c) CN600-15, (d) CN800-10, (e) CN800-12, (f) CN800-15, and (g) CN550.
